# Epidemiology of blood flukes (Digenea: Spirorchiidae) in sea turtles from Tyrrhenian and Adriatic Seas, off Italy

**DOI:** 10.1186/s13071-020-3922-9

**Published:** 2020-02-07

**Authors:** Mario Santoro, Erica Marchiori, Rudi Cassini, Michele Drigo, Doriana Iaccarino, Fabio Di Nocera, Barbara Degli Uberti, Giovanna De Luca, Marianna D’Amore, Cinzia Centelleghe, Mario Pietrobelli, Federica Marcer

**Affiliations:** 1 Department of Integrative Marine Ecology, Stazione Zoologica Anton Dohrn, Villa Comunale, 80121 Naples, Italy; 2 Department of Animal Medicine, Production and Health, University of Padova, 35020 Legnaro, Italy; 3 Istituto Zooprofilattico Sperimentale del Mezzogiorno, 80055 Portici, Italy; 4 Department of Comparative Biomedicine and Food Science, University of Padova, 35020 Legnaro, Italy

**Keywords:** *Caretta caretta*, *Chelonia mydas*, Green turtle, Loggerhead turtle, Spirorchiidiosis, *Hapalotrema mistroides*, *Neospirorchis* Neogen-11, Tyrrhenian Sea, Adriatic Sea, Mediterranean

## Abstract

**Background:**

The Spirorchiidae is a family of blood flukes parasitizing turtles. Spirorchiids may cause a wide range of inflammatory reactions in the vascular system of their host being frequently implicated with stranding and death of sea turtles worldwide. Recent studies revealed the presence of two spirorchiid species in the Mediterranean basin. Our study presents comparative epidemiological data of spirorchiid infections in loggerhead turtles (*Caretta caretta*) stranded during an eight-year period from Adriatic and Tyrrhenian Seas, and the first report of *Neospirorchis* Neogen-11 in a green turtle (*Chelonia mydas*).

**Methods:**

We screened a total of 319 carcasses of loggerhead turtles stranded from January 2011 to December 2018 along the Tyrrhenian coast (*n* = 111) and the north-western Adriatic coast (*n* = 208) of Italy using traditional (copromicroscopy and histopathology) and molecular assays. Three green turtles from the Tyrrhenian coast were also included in the study.

**Results:**

A total of 56 (17.5%) loggerhead turtles and one green turtle (33.3%) were found to be infected with spirorchiid flukes. Amplification, sequencing of the ITS2 region of the ribosomal RNA gene cluster and BLAST analysis confirmed the presence of *Hapalotrema mistroides* and *Neospirorchis* Neogen-11 in 51 (16.0%) and 24 (7.5%) loggerhead turtles, respectively, and *Neospirorchis* Neogen-11 in an infected green turtle. Differences in prevalence of infection between the two sampling areas were found.

**Conclusions:**

The risk of spirorchiid infection in the Tyrrhenian Sea is lower than in the Adriatic Sea and in general the risk of infection in the Mediterranean is lower than in other geographical locations. Differences in the prevalence of infection between the two sampling areas were related to the differences of regional habitats supporting different abundance of spirorchiid intermediate hosts. A systematic monitoring to evaluate the progress of the infection is recommended, as well as studies on the occurrence and distribution of spirorchiid species from other Mediterranean areas.
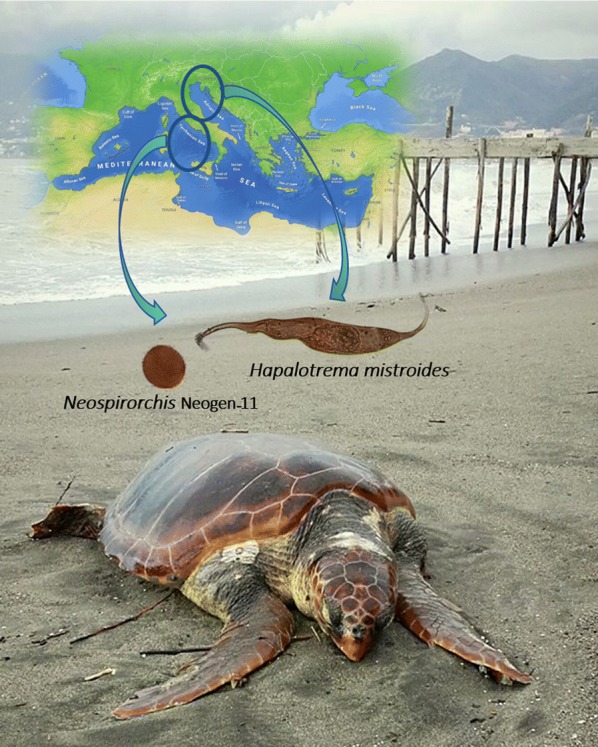

## Background

The Spirorchiidae Stunkard, 1921, is a family of blood flukes parasitizing the cardiovascular system of marine and freshwater turtles. To date, a total of 29 marine species belonging to 10 genera have been recognized [1–3]. Adult spirorchiids and their eggs may cause a wide range of inflammatory reactions in the vascular system of their host being frequently implicated with stranding and death of sea turtles worldwide [4–10]. Incidental findings of infection with or no associated diseases have also been described [11–15].

Except for the old reports of Monticelli [16] and Looss [17], published studies of spirorchiid infections from the Mediterranean are recent and limited to a survey from the north Adriatic Sea, where *Hapalotrema mistroides* (Monticelli, 1896), and *Neospirorchis* Neogen-11 were found in loggerhead turtles (*Caretta caretta*) [12], and a report on a fatal case associated with *H. mistroides* in an adult loggerhead turtle from the Tyrrhenian Sea [9].

In the last few years, knowledge concerning the biodiversity and epidemiology of spirorchiid flukes has been greatly enhanced through the use of molecular genetic approaches, together with traditional tools such as histopathological studies [2]. Using PCR and terminal restriction fragment length polymorphism (T-RFLP), Chapman et al. [18, 19] provided new data on spirorchiid flukes at the species level in Australian green turtles (*Chelonia mydas*) by analyses of tissues infected with their eggs. Using genetic targets, Stacy et al. [20], demonstrated the occurrence of at least 20 new distinct genotypes of *Neospirorchis* Price, 1934, correlated with host species and different tissue tropism in sea turtles of the Gulf of Mexico and Atlantic coasts of Florida (USA).

The knowledge on the life cycle of marine spirorchiids has also been enhanced by using molecular genetic tools. DNA of *Learedius learedi* Price, 1934 has been found in a single fissurellid gastropod *Fissurella nodosa* collected at a facility in Florida where green turtles were found to acquire *L. learedi* infections [21]. Recently, it has been found that the intermediate host responsible for an infection with a species of *Amphiorchis* Price, 1934 in captive hatched neonate loggerhead turtles housed in the Oceanogràfic Aquarium in Valencia (Spain) was the vermetid gastropod *Thylaeodus* cf. *rugulosus* [22]. Finally, during a recent survey of blood fluke larvae in polychaetes on the coast of South Carolina (USA), spirorchiid-like cercariae belonging to *Neospirorchis* Neogen-13 and *Neospirorchis* Neogen-14 (known to infect green turtles) were found to infect the polychaetes *Amphitrite ornata* (Terebellidae) and *Enoplobranchus sanguineus* (Polycirridae) [23].

Here, using traditional and molecular tools, we studied and compared for the first time the features of spirorchiid epidemiology in loggerhead turtles stranded during an eight-year period from two Mediterranean basins (i.e. Tyrrhenian and Adriatic Seas) addressing (i) the identification of spirorchiids to the species level; and (ii) the role of temporal, geographical and host-related factors on the occurrence and severity of spirorchiid infection of Mediterranean loggerhead turtles. Finally, a *Neospirorchis* sp. infection in a green turtle is reported for the first time from the Mediterranean Sea.

## Methods

### General data

A total of 319 carcasses of loggerhead turtles stranded from January 2011 to December 2018 along the Tyrrhenian coast of central and southern Italy (*n* = 111) and the north-western Adriatic coast (*n* = 208) was investigated for spirorchiid infections. Three green turtles collected in 2017 from the Tyrrhenian coast were also included in the study. For each turtle, whenever possible, the curved carapace length (CCL) and straight carapace length (SCL) were measured to the nearest cm and body weight was also registered. Sea turtles were categorized into the following age-classes [24]: juveniles, CCL ≤ 40 cm; sub-adults, CCL of 41–69 cm; and adults, CCL ≥ 70 cm. The body condition index (BCI) was calculated as body weight/SCL^3^ × 10^4^, and categorized as very good (> 1.20); good (1.19–1.10); average (1.09–1.0); or poor (< 1.0) [25]. In order to compare homogenous groups in terms of consistency, loggerhead turtles with a BCI classified as poor, average and good were assembled in a single group to be compared with the group classified as “very good” by statistical analysis. Whenever possible, the sex of the sea turtles was assessed at necropsy by direct observation of the gonads.

### *Post-mortem* examination

At the necropsy, all organs were grossly examined for adult spirorchiids, masses of their eggs and associated pathological changes. Samples of selected tissues (brain, heart, blood vessels, trachea, lungs, liver, gallbladder, kidneys, urinary bladder, pancreas, oviducts, oesophagus, stomach and intestine) and washing liquid from the body cavity of sea turtles were examined separately using a dissecting microscope [26, 27]. When present, adult parasites and their eggs were washed in saline solution and preserved in 70% ethanol or frozen at – 80 °C for morphological and molecular study, respectively.

### Direct examination for spirorchiid eggs

**Fig. 1:**
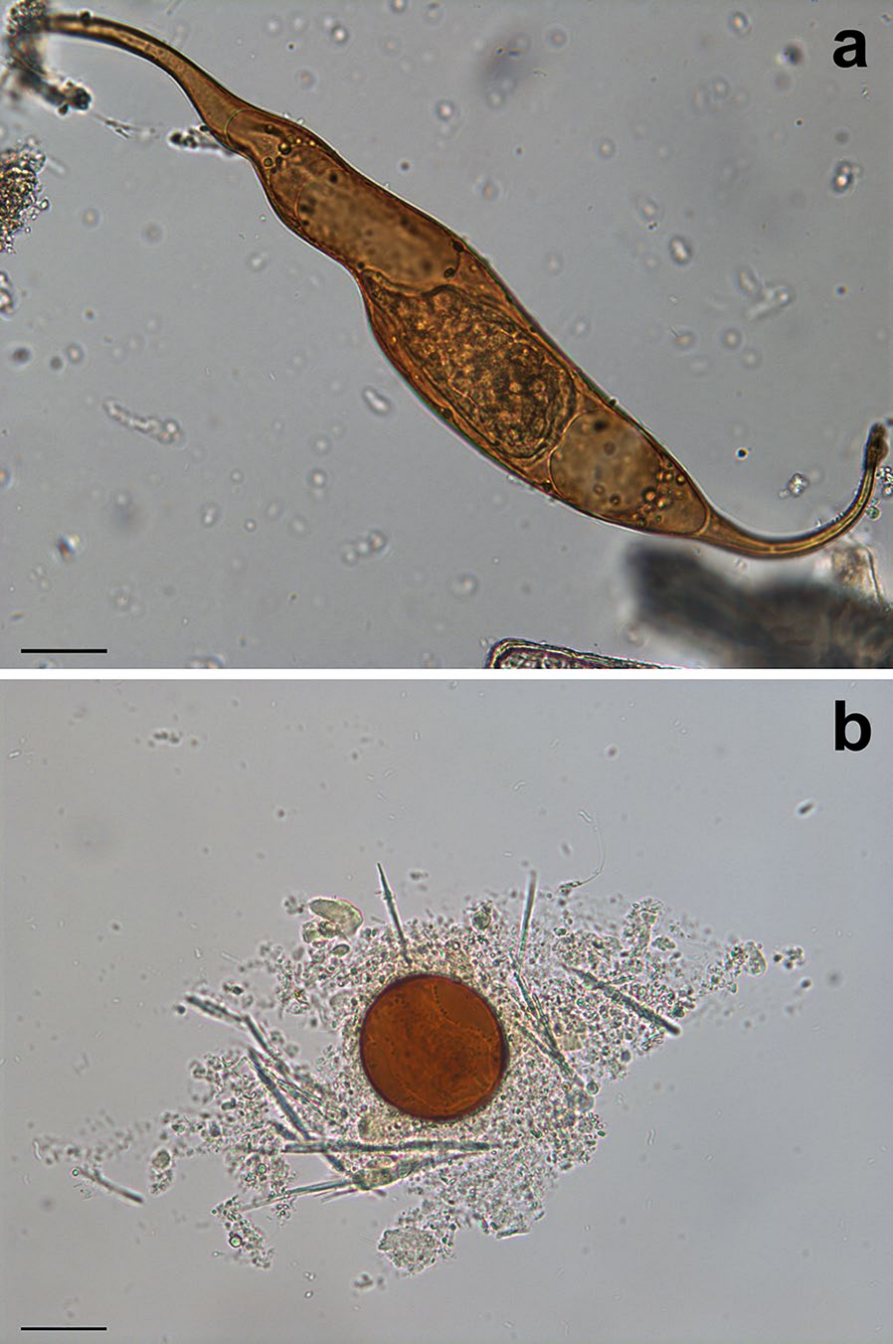
Eggs of *Hapalotrema mistroides* (**a**) and *Neospirorchis* Neogen-11 (**b**) from the feces of loggerhead turtles from the Mediterranean Sea. Note the miracidia still alive within the shell. *Scale-bars*: **a**, **b**, 30 μm

A standard sedimentation method was used to detect spirorchiid eggs from the feces (30 g) obtained in a *post-mortem* examination from the rectum. Egg detection was also performed for the spleen, as previously described by Marchiori et al. [12]. Briefly, a pre-weighed aliquot (2 g) of spleen sample was diced and homogenized in 10 ml tap water using a blender. The obtained fluid was centrifuged in a 15 ml tube at 2000× *rpm* for 5 min. After removal of the supernatant, a high-density solution (sodium nitrate, sodium thiosulphate and sucrose with a specific gravity of 1.45) was added and mixed with the sediment until the tube was full; a coverslip was left over the tube for 15 min and observed under a light microscope (magnification of 10×) for the detection of spirorchiid eggs. The eggs were then classified by their morphology as *Hapalotrema* (elongate with two polar processes) and *Neospirorchis* (round with no polar processes) types (Fig. 1). Samples of spleen and feces were chosen for direct examination of spirorchiid eggs because these represent the most important target sites where egg concentration occurs [6, 9, 12, 19, 20, 28].

### Histopathological study

Whenever possible, samples for histological examination of selected organs and tissues including brain, lungs, heart, stomach, intestine, pancreas, liver, spleen, urinary bladder and kidneys, were collected. Samples were fixed in 10% neutral phosphate-buffered formalin and processed by routine methods into paraffin blocks which were cut into 3 μm thick sections and stained with hematoxylin and eosin. Histologically, the severity of lesions associated with spirorchiid infection was scored for each examined tissue as follows: 0, absent; 1, mild (1 to several small, scattered granulomas per low power (20× magnification) field, each centered on ≤ 5 spirorchiid eggs); 2, moderate (1 to several granulomas centered on > 5 to 10 spirorchiid eggs per 20× magnification field, with some effacement of tissue architecture by granulomas); and 3, severe (numerous smaller granulomas, each centered on > 5 eggs, or large, often coalescing granulomas and/or sheets of inflammatory cells centered on ≥ 10 to hundreds of eggs, both with extensive effacement of tissue architecture) [9].

### Molecular identification

An aliquot of each organ and feces examined for spirorchiid flukes was collected and frozen at − 80 °C. When fecal, spleen or any histological sample was positive for spirorchiid eggs, DNA was extracted from frozen aliquots of those positive samples using the NucleoSpin^®^ Tissue Kit (Macherey-Nagel, Düren, Germany). Amplification of the internal transcribed spacer 2 (ITS2) region of the rDNA was carried out using the protocol described in Marchiori et al. [12] (length of expected fragment: 300 bp). The newly generated consensus sequences were compared with the data available on GenBank using the software BLAST [29].

### Statistical and risk factor analysis

The percentage of concordance and the *k* parameter were calculated for comparing the agreement between the qualitative copromicroscopic analysis and the examination of the spleen for parasite eggs [30].

A sea turtle was considered positive for spirorchiid infection when at least one of the two diagnostic procedures (copromicroscopic and spleen examinations) was positive. The overall prevalence of spirorchiid infection and the prevalence of *H. mistroides* and *Neospirorchis* Neogen-11 were calculated.

The prevalence of blood flukes (general for spirorchiids, and specific for *H. mistroides* and *Neospirorchis* Neogen-11) was investigated, building three multivariable logistic regression models including the following risk factors (predictors): sampling area (Adriatic Sea *vs* Tyrrhenian Sea), sampling year, age-class, BCI and sex. After the evaluation of autocorrelation and/or interaction between predictors, the output showing the higher parsimony was displayed. Sea turtles with missing information for one or more of the considered parameters were excluded from the analysis. A type 1 error probability of 0.05 was set as level of statistical significance. Statistical analyses were performed using the software IBM SPSS Statistics 25.

Descriptive statistics for the histopathological analysis, reporting the average and range of the scores assigned to infected loggerhead turtles, was performed to identify the organs and tissues most commonly affected by spirorchiid eggs.

## Results

### General data

CCL, age-class, sex, BCI, year of sampling, and collection site of loggerhead turtles are listed in Table 1. Prevalence of infection with spirorchiid flukes according to the geographical area, age-class, BCI, and sex of the loggerhead turtles are listed in Tables 2 and 3. At necropsy, gross findings related to spirorchiid infections were observed in a single loggerhead turtle infected with *H. mistroides* from the Tyrrhenian Sea (see Santoro et al. [9] for the case description). Mild to moderate arteritis of the major vessels was observed in seven loggerhead turtles from the Adriatic Sea, all of which were positive for *H. mistroides*. Serpiginous black stripes were observed in the intestinal mucosa of 12 loggerhead turtles and were further characterized by dissecting microscope as discrete masses of eggs of *Neospirorchis* type (see Marchiori et al. [12] for the description of lesions).

**Table 1:** Curved carapace length, age-class, body condition index, sex, and collection date of loggerhead turtles obtained from Adriatic and Tyrrhenian Seas

Area	CCL (cm)	Age-class				BCI					Sex			Sampling year							
	Mean ± SD	j	s-a	ad	nd	p	a	g	vg	nd	m	f	nd	2011	2012	2013	2014	2015	2016	2017	2018
A	53.5 ± 14.7	42	128	27	11	8	7	13	166	14	60	123	25	8	10	55	38	29	25	20	23
T	64.5 ± 10.6	10	64	35	2	9	4	9	75	14	48	55	8	4	6	5	11	14	9	31	31
Total	56.5 ± 14.9	52	192	62	13	17	11	22	241	28	108	178	33	12	16	60	49	43	34	51	54

*Abbreviations*: CCL, curved carapace length; j, juveniles; s-a, subadults; ad, adults; nd, not determined; BCI, body condition index; p, poor; a, average; g, good; vg, very good; m, male; f, female; SD, standard deviation; A, Adriatic Sea; T, Tyrrhenian Sea

**Table 2:** Prevalence of spirorchiid infections in loggerhead turtles according to the geographical area, age-class, body condition index, and sex

		*n*	Spirorchiids		*H. mistroides*		*Neospirorchis* Neogen-11	
			Positive	Prevalence (%)	Positive	Prevalence (%)	Positive	Prevalence (%)
Geographical area	Adriatic	208	43	20.7	41	19.7	20	9.6
	Tyrrhenian	111	13	11.7	10	9.0	4	3.6
Age-class	Juveniles	52	5	9.6	5	9.6	1	1.9
	Subadults	192	35	18.2	34	17.7	17	8.9
	Adults	62	16	25.8	12	19.4	6	9.7
BCI	Poor-average-good	50	8	16	7	14.0	5	10
	Very good	241	43	17.8	39	16.2	19	7.9
Sex	Male	108	17	15.7	16	14.8	7	6.5
	Female	178	33	18.5	29	16.3	13	7.3

*Abbreviations*: *n*, number examined; BCI, body condition index

**Table 3:** Prevalence of spirorchiid infections in loggerhead turtles according to the year of collection and geographical area

Area	Parasite species	2011	2012	2013	2014	2015	2016	2017	2018
		*n*/*N* (%)	*n*/*N* (%)	*n*/*N* (%)	*n*/*N* (%)	*n*/*N* (%)	*n*/*N* (%)	*n*/*N* (%)	*n*/*N* (%)
Adriatic	Spirorchiids	0/8 (0)	0/10 (0)	5/55 (9.1)	5/38 (13.1)	9/29 (31.0)	10/25 (40.0)	5/20 (25.0)	10/23 (39.1)
	*H. mistroides*	0/8 (0)	0/10 (0)	4/55 (7.2)	4/38 (10.5)	9/29 (31.0)	10/25 (40.0)	5/20 (25.0)	10/23 (39.1)
	*Neospirorchis* Neogen-11	0/8 (0)	0/10 (0)	1/55 (1.8)	3/38 (7.9)	3/29 (10.3)	7/25 (28.0)	0 (0)	6/23 (26.1)
Tyrrhenian	Spirorchiids	0/4 (0)	0/6 (0)	0/5 (0)	0/11 (0)	5/14 (35.7)	1/9 (11.1)	4/31 (12.9)	3/31 (9.7)
	*H. mistroides*	0/4 (0)	0/6 (0)	0/5 (0)	0/11 (0)	3/14 (21.4)	0/9 (0)	4/31 (12.9)	3/31 (9.7)
	*Neospirorchis* Neogen-11	0/4 (0)	0/6 (0)	0/5 (0)	0/11 (0)	2/14 (14.3)	1/9 (11.1)	1/31 (3.2)	0/31 (0)

*Abbreviations*: *n*, number infected; *N*, number examined

In general, 56 (17.5%) out 319 loggerhead turtles (including 13 from the Tyrrhenian Sea and 43 from the Adriatic Sea), were found to be infected with spirorchiid flukes through direct examination for eggs in the feces and/or spleen. As for detection of *H. mistroides* eggs, the copromicroscopic analysis showed an excellent agreement with the examination of the spleen for eggs (concordance 95.9%, *k* parameter: 0.830) whereas there was a fair agreement in the case of *Neospirorchis* sp. (concordance 95.3%, *k* parameter: 0.496). Eggs of *Hapalotrema* and *Neospirorchis* types were found in 51 (16.0%) and 24 (7.5%) loggerhead turtles, respectively; mixed infections were found in 19 loggerhead turtles (5.9%) (Table 2). Adult flukes (intensity range: 1–41) were recovered from heart, great vessels and spleen of eight loggerhead turtles (including one from the Tyrrhenian Sea and seven from the Adriatic Sea), and were all morphologically identified as *H. mistroides*.

One out of three green turtles was found to be positive for eggs of *Neospirorchis* type by both direct examination of feces and spleen. The positive green turtle was an adult male (CCL: 70.1 cm) found stranded on 9 September 2017 on the beach of Mondragone (Caserta municipality).

### Histopathological study

Overall, 217 sea turtles were sampled for histology. Sea turtles which were negative for spirorchiid egg detection *via* coprological and/or spleen examination were also negative in a histological examination of all sampled organs (*n* = 164). In loggerhead turtles positive to *Hapalotrema*, the specific egg type was observed in histological sections of the heart (2/19, 10.5%), urinary bladder (2/9, 22.2%), pancreas (3/11, 27.3%), liver (6/20, 30%), kidneys (6/20, 30%), stomach (5/15, 33.3%), lungs (8/21, 38.1%), intestine (16/24, 66.7%), and spleen (35/40, 87.5%). Similarly, the *Neospirorchis* egg type was observed in histological sections of the liver (1/10, 10%), spleen (9/24, 37.5%) and intestine (19/20, 95%). In the positive green turtle, eggs of *Neospirorchis* type were observed in histological sections of the intestine, spleen and liver.

The severity of lesions expressed as average severity score (ASS) associated with spirorchiid infection for each organ is listed in Table 4. The spleen was the organ with a higher ASS value, followed by the intestine (Table 4).

**Table 4:** Tissue samples of loggerhead turtles studied by histopathology for spirorchiid infections according to the geographical area

Area		Liver	Pancreas	Stomach	Intestine	Heart	Lungs	Kidneys	Urinary bladder	Spleen
Adriatic	No. tested	13	3	9	18	12	14	13	2	32
	ASS	0.31 (0–1)	0.33 (0–1)	0.33 (0–1)	1.33 (0–3)	0.08 (0–1)	0.43 (0–2)	0.38 (0–1)	0.50 (0–1)	0.97 (0–2)
	SD	0.48	0.58	0.50	0.84	0.29	0.65	0.51	0.71	0.59
Tyrrhenian	No. tested	11	11	11	11	11	11	11	10	12
	ASS	0.27 (0–2)	0.64 (0–3)	0.27 (0–2)	0.45 (0–3)	0.18 (0–2)	0.36 (0–2)	0.18 (0–2)	0.10 (0–1)	2.08 (1–3)
	SD	0.65	1.21	0.65	0.93	0.60	0.67	0.60	0.32	0.90
Total	No. tested	24	14	20	29	23	25	24	12	44
	ASS	0.29 (0–2)	0.57 (0–3)	0.30 (0–2)	1.00 (0–3)	0.13 (0–2)	0.40 (0–2)	0.29 (0–2)	0.17 (0–1)	1.27 (0–3)
	SD	0.55	1.09	0.57	0.96	0.46	0.65	0.55	0.39	0.85

*Abbreviations:* ASS, average severity score; SD, standard deviation

*Notes*: Only tissues positive at least one time were here included. Values of severity score (0–3) are expressed as average severity score, with range reported in parentheses

### Molecular identification

Amplification and sequencing of the ITS2 region of samples of spirorchiid eggs and adult flukes resulted in 80 sequences which were of good enough quality for analysis. BLAST analyses indicated that 52 sequences of eggs were of the *Hapalotrema* type and 8 sequences of adult flukes found in 48 loggerhead turtles were identical to a *H. mistroides* sequence (GenBank: KY499798). These newly generated sequences were deposited in the GenBank database under accession numbers MN587705-MN587710. A total of 20 sequences of the *Neospirorchis* egg type, found in 19 loggerhead turtles and one green turtle, were identical to a *Neospirorchis* Neogen-11 sequence (GenBank: LT617053). These newly generated sequences, including the isolate from the green turtle, were deposited in the GenBank database under the accession numbers MN587699-MN587704.

### Statistical and risk factor analysis

**Fig. 2:**
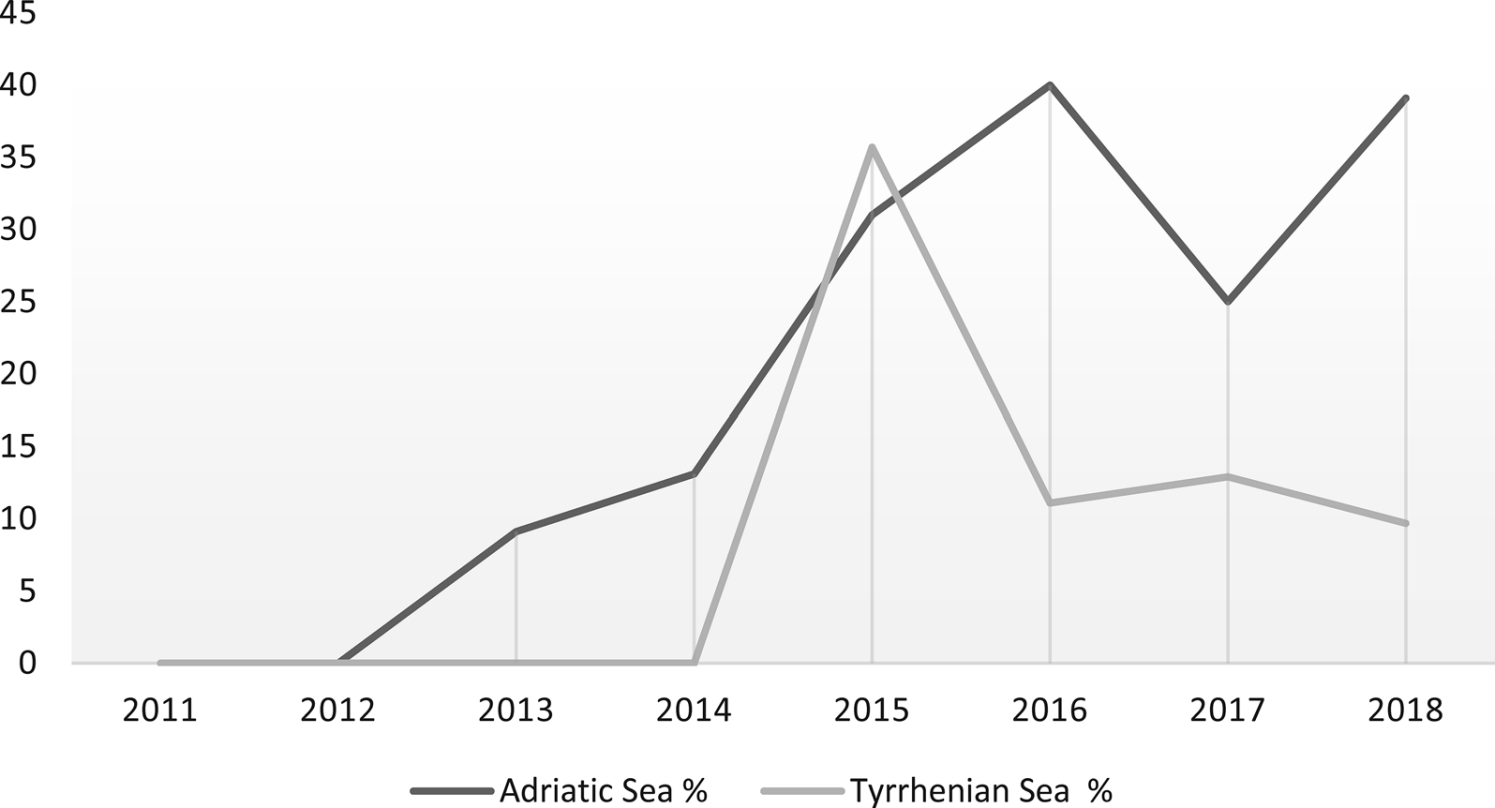
Prevalence of spirorchiidiasis in loggerhead turtles from the Adriatic and Tyrrhenian Seas according to the year of sampling

The trend of prevalence of infection along with years of sampling for both *H. mistroides* and *Neospirorchis* Neogen-11 is reported in Table 3 and Fig. 2, for both Mediterranean basins.

The influence of different predictors in determining the positivity for spirorchiids was investigated by means of a multivariable approach, including a total of 281 loggerhead turtles, since not all information was available for 28 subjects. The multivariable logistic regression models demonstrated that the Adriatic Sea represents a risk factor for spirorchid infection (OR = 5.118) and also for both *H. mistroides* (OR = 7.344) and *Neospirorchis* Neogen-11 (OR = 5.893) (Table 5). Besides, the prevalence was significantly increasing from 2011 onward for spirorchiids in general and individually for both species. Finally, a significant increase in prevalence along with age-class was also observed for spirorchiids and *Neospirorchis* Neogen-11, whereas *H. mistroides* prevalence in adult animals was not significantly different from values in younger animals. There was no significant association between prevalence of infection and sex or BCI (data not shown).

**Table 5:** Results of the multivariable logistic regression models in loggerhead turtles (*n* = 281) according to sampling area, sampling year and host age-class

Parasite species	Factor	Description	Odds ratio	95% CI	*P*-value
Spirorchiids	Sampling area	Adriatic *vs* Tyrrhenian	5.118	2.220–11.801	< 0.001
	Sampling year	From 2011 to 2018	1.407	1.178–1.680	< 0.001
	Age-class	Increasing age	2.048	1.166–3.600	0.013
*H. mistroides*	Sampling area	Adriatic *vs* Tyrrhenian	7.344	2.875–18.759	< 0.001
	Sampling year	From 2011 to 2018	1.500	1.242–1.811	< 0.001
	Age-class	Increasing age	1.638	0.913–2.941	0.098
*Neospirorchis* Neogen-11	Sampling area	Adriatic *vs* Tyrrhenian	5.893	1.789–19.414	0.004
	Sampling year	From 2011 to 2018	1.452	1.141–1.850	0.002
	Age-class	Increasing age	2.166	1.004–4.672	0.049

*Abbreviations*: CI, confidence interval

## Discussion

This study represents the most extensive molecular study on blood flukes of sea turtles. Because of the large number of sea turtles included, we performed molecular analyses only on fecal and tissue samples that tested positive to the coprological and histopathological analyses. The values of concordance among fecal, spleen and histological analyses suggest trustworthy results regarding the actual situation of spirorchiid prevalence; however, consideration should be given to the possibility that a small number positive cases have not been detected [18, 19].

Our results demonstrate the occurrence of only two spirorchiid species (*H. mistroides* and *Neospirorchis* Neogen-11) in sea turtles from both Mediterranean basins under study. Comparison between sequences of the ITS2 fragment of *H. mistroides* and *Neospirorchis* Neogen-11 revealed the lack of genetic variation for this marker between the fluke populations from sea turtles from the Mediterranean and Atlantic waters of Florida for both species. Previous studies have also shown the identity among sequences of the *28S* gene from Mediterranean and Florida isolates of *H. mistroides* and *Neospirorchis* Neogen-11 [9, 12]. Phylogeographical studies of the crustacean *Chelonibia testudinaria*, an obligate commensal commonly observed among epibiotic fauna of loggerhead turtles, revealed low levels of genetic divergence between isolates from off Florida and Greece [31]. These authors suggested that migration of juvenile loggerhead turtles from the Western Atlantic coasts to the Mediterranean could maintain gene flow among the two populations of coronulid barnacles, and is also responsible for their range expansion from the Western Atlantic into the Mediterranean [31]. Nevertheless, further analyses are needed and encouraged to clarify whether a similar scenario could also apply to blood flukes of sea turtles, given that the ecology of parasites is deeply divergent and that molecular data for other loci (e.g. *cox*1) are still needed to thoroughly analyse the intraspecific variation.

Although at the *post-mortem* examination, spirorchiid infection was observed in 17.5% of loggerhead turtles and in one out of three green turtles, spirorchiid infection has been considered as the primary cause of death only in a single loggerhead turtle from the Tyrrhenian Sea where *H. mistroides* infection occurred [9]. We assume that most of the infections with spirorchiids were incidental within the two sampled populations. The majority of the sea turtles were classified with a “good” BCI thus supporting the hypothesis that they likely died due to acute lethal events linked to other causes. However, whether spirorchiids may have indirectly impacted the fitness and longevity of infected sea turtles remains unknown. Our results agree with studies on green turtles from Hawaii [11] and Costa Rica [13, 14, 28] where although pathological changes by spirorchiid infection were commonly observed, those were rarely considered non-compatible with the life of the host. In contrast, fatal infections have been reported in Florida [5, 6], Australia [4, 7, 8] and Brazil [10].

The impact of a parasite on its host may depend on geographical variables, and may also change over time [32] as demonstrated by the increase in pathogenic effects of spirorchiidiasis in stranded green turtles in Queensland (Australia) over the past two decades [2, 4, 5, 8, 19]. Therefore, a systematic monitoring of spirorchiid infections has been recommended [2].

The diversity of geographical and ecological features of the two studied basins could also account for the difference in the prevalence of infection between loggerhead turtles from Tyrrhenian and Adriatic Seas. A difference in prevalence of infection was also recently found for the anisakid nematode *Sulcascaris sulcata* (Rudolphi, 1819) [33]. The northern area of the Adriatic Sea occupies the flooded seaward extension of the Po Plain and reaches an average depth of about 35 m with a sandy or muddy bottom. It is the most extensive continental shelf of the entire Mediterranean Sea, with high macrofaunal density dominated by sedentary invertebrates. This basin hosts one of the largest neritic foraging habitats for loggerhead turtles in the Mediterranean [34, 35]. A high degree of biodiversity could reasonably support a greater abundance of one or more intermediate hosts for both *H. mistroides* and *Neospirorchis* Neogen-11 in the Adriatic benthic communities. The unique features of the northern Adriatic ecosystem reasonably support a higher probability for the loggerhead turtles to become infected with parasites that use invertebrate intermediate hosts to complete their life cycle [33]. Besides, bathymetric and hydrobiological characteristics of such an enclosed basin are also responsible for higher water temperatures throughout the summer months, which could hypothetically in turn influence the ecology of the parasites enhancing the speed of development of larval stages [36].

We also found a difference in prevalence of spirorchiid infection according to the year of sampling for both sampling basins. We recorded the first case of infection from the Adriatic Sea in December 2009 [12] and observed an increasing trend of prevalence from 2014 to 2015 which remained stable throughout the successive years (Fig. 2, Table 4). In contrast, we recorded the first case of an infected turtle from the Tyrrhenian Sea in June 2015 [9] with a peak of infection (35.7%) in the same year; since then the prevalence has settled at levels of 9.7–12.9% (Fig. 2, Table 4). The reasons for these variations over time remain poorly understood. They could be related to environmental seasonal factors across the years in which the peaks were observed. However, the explanation for this finding is impaired by the lack of data on the biological cycle of the parasites.

Regarding the increasing trends of prevalence along with age, Stacy et al. [6] observed that in Florida, larger loggerhead and green turtles had greater intensities and prevalence of spirorchiid infection associated with pathological changes than smaller turtles. Only 1 out of 46 juveniles was found to be infected with *L. learedi* and none were found infected with *Hapalotrema* spp. Infection with *Hapalotrema*/*Learedius* was also less frequent in immature loggerhead turtles than in adults [6]. The burden of *Neospirorchis* spp. appeared to increase with carapace length in both green and loggerhead turtles [6]. Similarly, necropsies of sea turtle pelagic stages including 88 loggerhead turtles, three green turtles, two leatherback turtles (*Dermochelys coriacea*) and 1860 loggerhead turtles, stranded on the coasts of the Canary Islands (Spain), did not show evidence for spirorchiid infection [37, 38]. Evidence from those studies and most of our results supports the hypothesis that sea turtles primarily acquire spirorchiid infection while foraging in shallower and coastal waters where potential intermediate hosts should be abundant [2, 39]. Moreover, spirorchiid eggs are known to accumulate in the organs over time, probably explaining the increasing trend of positivity along with the increasing age of the sea turtle.

In a recent survey of *Neospirorchis* in sea turtles from the coasts of Florida, Stacy et al. [20] identified at least 20 different genotypes (numbered Neogen-1 through -20), most of which exhibited host and site specificity. These authors found *Neospirorchis* Neogen-11 (flukes and their eggs) exclusively in enteric submucosal vessels of loggerhead turtles, suggesting strong host specificity and the enteric submucosa as the primary site predilection for this genotype.

In the present study, no adults of *Neospirorchis* Neogen-11 were found. Big clusters of densely aggregated eggs in the shape of black stripes were observed with higher frequencies in the intestine. The pattern of distribution of eggs in this tissue confirms that it represents the primary site of deposition by mature parasites likely to be living in enteric vessels [20]. Nevertheless, eggs of *Neospirorchis* Neogen-11 were also found in the spleen and liver as scattered elements, probably embolized through hematic circulation. Moreover, we report for the first time the occurrence of *Neospirorchis* Neogen-11 in a green turtle. To date, there is only one historical record of spirorchiid infection (*H. mistroides*) in green turtles from the Mediterranean Sea [17].

According to Chapman et al. [2], an aspect of the marine spirorchiid life cycle yet to be thoroughly explored is the route of egg elimination from its definitive host. Three main possible shedding pathways have been hypothesised, including through feces, expectoration, and through *post-mortem* decomposition or scavenging of a carcass with egg dispersion [2]. In all positive turtles we were able to collect the eggs of both *H. mistroides* and *Neospirorchis* Neogen-11 from the spleen and feces. In several of these carcasses we obtained an enormous quantity of eggs containing live miracidium (Fig. 1) from both spleen and feces. This suggests, that at least for *H. mistroides* and *Neospirorchis* Neogen-11, viable eggs might be shed through the feces or dispersion after *post-mortem* decomposition.

## Conclusions

To the best of our knowledge, this is the first comparative study on molecular epidemiology of spirorchiid infection in loggerhead turtles from the Mediterranean basin, and the first report of *Neospirorchis* Neogen-11 in a green turtle. Although it appears that the risk of spirorchiid infection in the Tyrrhenian is lower than in the Adriatic, and a general trend suggests that the risk of infection in the Mediterranean Sea is lower than in other geographical areas [2], a systematic monitoring to evaluate the progress of the infection is recommended, as well as studies on the occurrence and distribution of spirorchiid species from other Mediterranean areas. Studies are currently under way to identify, according to its poorly known life cycle [21–23], which invertebrate species are acting as intermediate hosts for these parasites in the Mediterranean. Finally, genetic variability of the spirorchiid species isolated in the Mediterranean and Western Atlantic should be thoroughly examined and compared to evaluate the possible dissemination of blood flukes among the two marine basins following migratory pathways of sea turtles.

## Data Availability

The data supporting the conclusions of this article are included within the article.

## Abbreviations

PCR: polymerase chain reaction
T-RFLP: terminal restriction fragment length polymorphism
CCL: curved carapace length
SCL: straight carapace length
BCI: body condition index
ITS2: internal transcribed spacer 2
ASS: average severity score

## References

[1] Platt TR, Gibson DT, Jones A, Bray RA (2002). Family spirorchiidae Stunkard, 1921. Keys to the Trematoda.

[2] Chapman PA, Cribb TH, Flint M, Traub RJ, Blair D, Kyaw-Tanner MT, Mills PC (2019). Spirorchiidiasis in marine turtles: the current state of knowledge. Dis Aquat Org.

[3] Werneck MR, Da Silva RJ (2015). Checklist of sea turtles endohelminth in Neotropical region. Helminthologia..

[4] Gordon AN, Kelly WR, Cribb TH (1998). Lesions caused by cardiovascular flukes (Digenea: Spirorchidae) in stranded green turtles (*Chelonia mydas*). Vet Pathol..

[5] Jacobson ER, Homer BL, Stacy BA, Greiner EC, Szabo NJ, Chrisman CL (2006). Neurological disease in wild loggerhead sea turtles *Caretta caretta*. Dis Aquat Org..

[6] Stacy BA, Foley AM, Greiner E, Herbst LH, Bolten AB, Klein PA (2010). Spirorchiidiasis in stranded loggerhead *Caretta caretta* and green turtles *Chelonia mydas* in Florida (USA): host pathology and significance. Dis Aquat Org..

[7] Flint M, Patterson-Kane JC, Limpus CJ, Mills PC (2010). Health surveillance of stranded green turtles in southern Queensland, Australia (2006–2009): an epidemiological analysis of causes of disease and mortality. EcoHealth..

[8] Flint M, Eden PA, Limpus CJ, Owen H, Gaus C, Mills P (2015). Clinical and pathological findings in green turtles (*Chelonia mydas*) from Gladstone, Queensland: investigations of a stranding epidemic. EcoHealth..

[9] Santoro M, Di Nocera F, Iaccarino D, Lawton SP, Cerrone A, Degli Uberti B (2017). Pathology and molecular analysis of *Hapalotrema mistroides* (Digenea: Spirorchiidae) infecting a Mediterranean loggerhead turtle *Caretta caretta*. Dis Aquat Org..

[10] Jerdy H, Werneck M, Goldberg D, Baldassin P, Feriolli R, Maranho A (2020). Ocular spirorchiidiosis in sea turtles from Brazil. J Helminthol..

[11] Work TM, Balazs GH, Summers TM, Hapdei JR, Tagarino AP (2015). Causes of mortality in green turtles from Hawaii and the insular Pacific exclusive of fibropapillomatosis. Dis Aquat Org..

[12] Marchiori E, Negrisolo E, Cassini R, Garofalo L, Poppi L, Tessarin C, Marcer F (2017). Cardiovascular flukes (Trematoda: Spirorchiidae) in *Caretta caretta* Linnaeus, 1758 from the Mediterranean Sea. Parasites Vectors..

[13] Santoro M, Morales JA (2007). Some digenetic trematodes of the olive ridley sea turtle, *Lepidochelys olivacea* (Testudines, Cheloniidae) in Costa Rica. Helminthologia.

[14] Santoro M, Morales JA, Bolanos F, Chaves G, De Stefano M (2015). Helminths of hawksbill turtle (*Eretmochelys imbricata*) from the Pacific coast of Costa Rica. Helminthologia..

[15] Jerdy H, Ribeiro RB, Silva MA, Medina RM, Werneck MR, Carvalho ECQ (2016). Spirorchiid infection in the olive ridley turtle, *Lepidochelys olivacea* (Eschscholtz, 1829) (Testudines: Cheloniidae) from Brazil. J Parasitol..

[16] Monticelli FS (1896). Di un ematozoo della *Thalassochelys caretta* Linn. Int Mschr Anat Physiol..

[17] Looss A (1899). Weitere Beiträge zur Kenntniss der Trematoden-Fauna Aegyptens, zugleich Versuch einer natürlichen Gliederung des Genus *Distomum* Retzius. Zool Jahrb Abt Systematik, Ökologie und Geographie der Tiere..

[18] Chapman PA, Traub RJ, Kyaw-Tanner MT, Owen H, Flint M, Cribb TH, Mills PC (2016). Terminal restriction fragment length polymorphism for the identification of spirorchiid ova in tissues from the green sea turtle, *Chelonia mydas*. PLoS ONE.

[19] Chapman PA, Owen H, Flint M, Soares Magalhães RJ, Traub RJ, Cribb TH (2017). Molecular epidemiology and pathology of spirorchiid infection in green sea turtles (*Chelonia mydas*). Int J Parasitol Parasites Wildl..

[20] Stacy BA, Chapman PA, Foley AM, Greiner EC, Herbst HL, Bolten AB (2017). Evidence of diversity, site and host specificity of sea turtle blood flukes (Digenea: Schistosomatoidea: ‘Spirorchiidae’): a molecular prospecting study. J Parasitol..

[21] Stacy BA, Frankovich T, Greiner E, Alleman AR, Herbst LH, Klein P (2010). Detection of spirorchiid trematodes in gastropod tissues by polymerase chain reaction: preliminary identification of an intermediate host of *Learedius learedi*. J Parasitol..

[22] Cribb TH, Crespo-Picazo JL, Cutmore SC, Stacy BA, Chapman PA, García-Párraga D (2017). Elucidation of the first definitively identified life cycle for a marine turtle blood fluke (Trematoda: Spirorchiidae) enables informed control. Int J Parasitol..

[23] de Buron I, Colon BL, Siegel SV, Oberstaller J, Rivero A, Kyle DE (2018). First evidence of polychaete intermediate hosts for *Neospirorchis* spp. marine turtle blood flukes (Trematoda: Spirorchiidae). Int J Parasitol..

[24] Bolten AB, Eckert KL, Bjorndal KA, Abreu-Grobois FA, Donnelly M (1999). Techniques for measuring sea turtles. Research and management techniques for the conservation of sea turtles.

[25] Thomson JA, Burkholder D, Heithaus MR, Dill LM (2009). Validation of a rapid visual-assessment technique for categorizing the body condition of green turtles (*Chelonia mydas*) in the field. Copeia..

[26] Greiner EC, Forrester DJ, Jacobson ER (1980). Helminths of mariculture-reared green turtles (*Chelonia mydas mydas*) from Grand Cayman, British West Indies. Proc Helminthol Soc Wash..

[27] Snyder SD, Clopton RE (2005). New methods for the collection and preservation of spirorchiid trematodes and polystomatid monogeneans from turtles. Comp Parasitol..

[28] Santoro M, Morales JA, Rodriguez-Ortiz B (2007). Spirorchiidiosis (Digenea: Spirorchiidae) and lesions associated with parasites in Caribbean green turtles (*Chelonia mydas*). Vet Rec..

[29] Altschul SF, Gish W, Miller W, Myers EW, Lipman DJ (1990). Basic local alignment search tool. J Mol Biol..

[30] Landis JR, Koch GG (1977). The measurement of observer agreement for categorical data. Biometrics..

[31] Rawson PD, Macnamee R, Frick MG, Williams KL (2003). Phylogeography of the coronulid barnacle, *Chelonibia testudinaria*, from loggerhead sea turtles, *Caretta caretta*. Mol Ecol..

[32] Deem SL, Karesh WB, Weisman W (2001). Putting theory into practice: wildlife health in conservation. Conserv Biol..

[33] Santoro M, Marchiori E, Iaccarino D, Uberti BD, Cassini R, Di Nocera F (2019). Epidemiology of *Sulcascaris sulcata* (Nematoda: Anisakidae) ulcerous gastritis in the Mediterranean loggerhead sea turtle (*Caretta caretta*). Parasitol Res..

[34] Margaritoulis D, Argano R, Baran I, Bentivegna F, Bradai MN, Camiñas JA, Bolten AB, Witherington BE (2003). Loggerhead turtles in the Mediterranean Sea: present knowledge and conservation perspectives. Loggerhead sea turtles.

[35] Lazar B, Margaritoulis D, Tvrtković N (2004). Tag recoveries of loggerhead sea turtles *Caretta caretta* in the eastern Adriatic Sea: implications for conservation. J Mar Biol Assoc UK.

[36] Holliman RB (1971). Ecological observations on two species of spirorchid trematodes. Am Midl Nat..

[37] Orós J, Torrent A, Calabuig P, Déniz S (2005). Diseases and causes of mortality among sea turtles stranded in the Canary Islands, Spain (1998–2001). Dis Aquat Org.

[38] Orós J, Montesdeoca N, Camacho M, Arencibia A, Calabuig P (2016). Causes of stranding and mortality, and final disposition of loggerhead sea turtles (*Caretta caretta*) admitted to a wildlife rehabilitation center in Gran Canaria Island, Spain (1998–2014): a long-term retrospective study. PLoS ONE.

[39] Work TM, Balazs GH, Schumacher JL, Amarisa M (2005). Epizootiology of spirorchiid infection in green turtles (*Chelonia mydas*) in Hawaii. J Parasitol..

